# Results of an international survey of SunCHECK Patient users: consensus and benchmark data for plan verification, pre-treatment quality assurance, and in vivo dosimetry tolerances

**DOI:** 10.1093/bjr/tqag166

**Published:** 2026-07-29

**Authors:** Aisling Haughey, Robert Biggar, Rhydian Caines, Stephen Moloney, James Earley, Paul Doolan

**Affiliations:** North West Cancer Centre, WHSCT, Derry, BT47 6SB, United Kingdom; Royal Devon University Healthcare NHS Foundation Trust, Exeter, EX2 5DW, United Kingdom; University of Liverpool and The Clatterbridge Cancer Centre NHS Foundation Trust, Liverpool, L7 8YA, United Kingdom; University Hospitals Dorset NHS Foundation Trust, Poole, BH15 2JB, United Kingdom; Royal Surrey NHS Foundation Trust, Guildford, GU2 7XX, United Kingdom; German Oncology Centre, Limassol, 4108, Cyprus

**Keywords:** EPID, in vivo dosimetry, patient QA

## Abstract

**Objectives:**

Electronic portal imaging devices (EPID) are well suited to radiotherapy in vivo dosimetry (IVD), but limited guidance remains a barrier to routine clinical use. This SunCHECK (Sun Nuclear Corporation) user survey captured real-world configurations and tolerances for EPID-based patient-specific verification, as well as other aspects of patient-related QA, to establish consensus benchmark data and inform guidance for new and existing users.

**Methods:**

A structured survey was distributed to SunCHECK Patient users across the United Kingdom and Europe. Respondents reported current clinical practice for treatment plan verification, patient-specific quality assurance (PSQA), and IVD, including calibration methods, analysis modes, and investigation tolerances.

**Results:**

Sixty-four responses were received from 18 countries. Linear accelerator manufacturer, treatment planning algorithm, and level of SunCHECK experience had minimal influence on applied tolerances. Point dose difference is no longer widely used as a pass-fail metric. Most centers use gamma analysis for calibration with global normalization, a 10% threshold, and a 95% pass rate. Plan verification and PSQA are almost universally assessed using gamma criteria of 3%/3 mm with a 95% passing tolerance. IVD tolerances fall into 2 main approaches: gamma criteria of 10%/5 mm for detecting gross errors, and tighter criteria of either 3%/3 mm or 5%/5 mm for identifying smaller, clinically relevant discrepancies.

**Conclusions:**

Despite extensive configurability within SunCHECK, there is strong consistency across centers in selected settings and investigation tolerances. These benchmark data provide practical guidance for reviewing and optimizing local configurations.

**Advances in knowledge:**

This work provides consensus-based guidance for configuring EPID IVD systems to reliably identify treatment delivery inconsistencies in clinical practice.

## Introduction

Radiotherapy treatment techniques are continuously increasing in conformality^1^ and the associated increase in complexity of treatment delivery must be matched in the ability to verify that the dose delivered to patients is geometrically and dosimetrically accurate. This is mandated by UK^2^ and European legislation^3^ and reinforced in national guidance documents.^4^ A traditional point dose check is not considered adequate for complex treatment deliveries,^5^ which has resulted in the advancement of treatment plan verification options. Electronic portal imaging devices (EPID) are widely available on commercial linear accelerators (linacs) and have been shown to be dosimetrically equivalent to conventional detector arrays for IMRT and VMAT QA.^6^

Publications in the United Kingdom^5–7^ have recommended that all centers should have protocols in place for performing in vivo dosimetry (IVD). MacDougall et al^5^ demonstrated that, by 2017, 73% of UK centers had an IVD program. EPID dosimetry was seen to offer the most potential and van Elmpt et al^8^ outlined potential errors that could be detected by EPID dosimetry. EPID IVD (EIVD) has been growing in use as it has become widely available through commercial systems. A 2020 UK survey^9^ indicated that 37% of UK radiotherapy centers are now using EIVD, and 84% recognize that it is an optimal IVD solution. The 2020 survey indicated that the main barriers to implementation of EIVD were the need for automation and lack of guidance for commissioning and appropriate system tolerances.

The International Atomic Energy Agency (IAEA) produced a report^10^ for the development of IVD in radiotherapy, however, it pre-dated the routine use of EVID and is therefore not referred to in this work. The AAPM Task Group 218 (TG218)^11^ report outlines recommendations for tolerances for pretreatment patient-specific quality assurance (PSQA) and advises individual centers on a process to determine appropriate local tolerances. However, these recommendations also exclude EIVD.

SunCHECK Patient (Sun Nuclear Corporation, Melbourne, FL, USA) is a commercially available platform that can perform dosimetry verification of treatment plans, PSQA and EIVD.^12^ Within the SunCHECK platform, there are several components with different uses (further details can be found at the SunCHECK website^13^). Secondary dose calculations for verification of treatment plans are performed in the DoseCHECK module, which compares the 3D dose distribution from the treatment planning system (TPS) to its own calculation. Pretreatment PSQA is performed in the Fraction 0 module, where a portal 2D dosimetry image, acquired without the patient present, is compared to a predicted image.^14^ A 3D dosimetric comparison, calculated on the planning CT, is also possible using linac log file data. IVD/online treatment monitoring is performed in the Fraction N module. This may be used to acquire a transit 2D dosimetric portal image during treatment for subsequent comparison with a predicted image, or with a reference image from a previous fraction. Additionally, data from the linac log file may be used in a 3D dose calculation on either the planning CT or CBCT. Alternatively, in both the Fraction Zero (F0) and Fraction N (FN) modules, EPID images acquired in cine mode may be used to determine MLC and collimator positions, which are then combined with other data from the linac log file and in a 3D dose calculation. This mode is termed “Epilog.” All dose calculations are performed using the Sun Nuclear Dose Calculator (SDC) algorithm,^15^ which is a superposition/convolution algorithm that includes a Total Energy Released per unit MAss (TERMA) calculation using medium based attenuation coefficient values. Each event is analyzed automatically according to pre-defined templates. The templates can be either “Custom” templates with site- or dose-specific tolerances defined by the user, or “Universal” templates which apply generic QA criteria. Both types of analysis templates are editable. If universal templates are chosen, results are calculated simultaneously with 3 sets of criteria, supporting centers that wish to employ separate warning and suspension levels.

When 2D portal dose images are required, this is accomplished through a calibration that relates the imaging signal acquired to the dose to water at the plane of the detector. The calibration process consists of the delivery of a series of rectangular fields of varying widths and positions to the EPID, which is automatically processed by SunCHECK to build a kernel which characterizes the panel response.

Day-to-day workflow is largely automated using DICOM query/retrieve and background processing of dosimetric events, but the system is also highly customizable with multiple steps and decision points required to first commission the system for use in clinics, which could present a challenge when there is relatively limited published guidance. Figure 1 illustrates an Ishikawa Fishbone diagram, indicating the many factors a new user must consider when implementing SunCHECK Patient.

**Figure 1:**
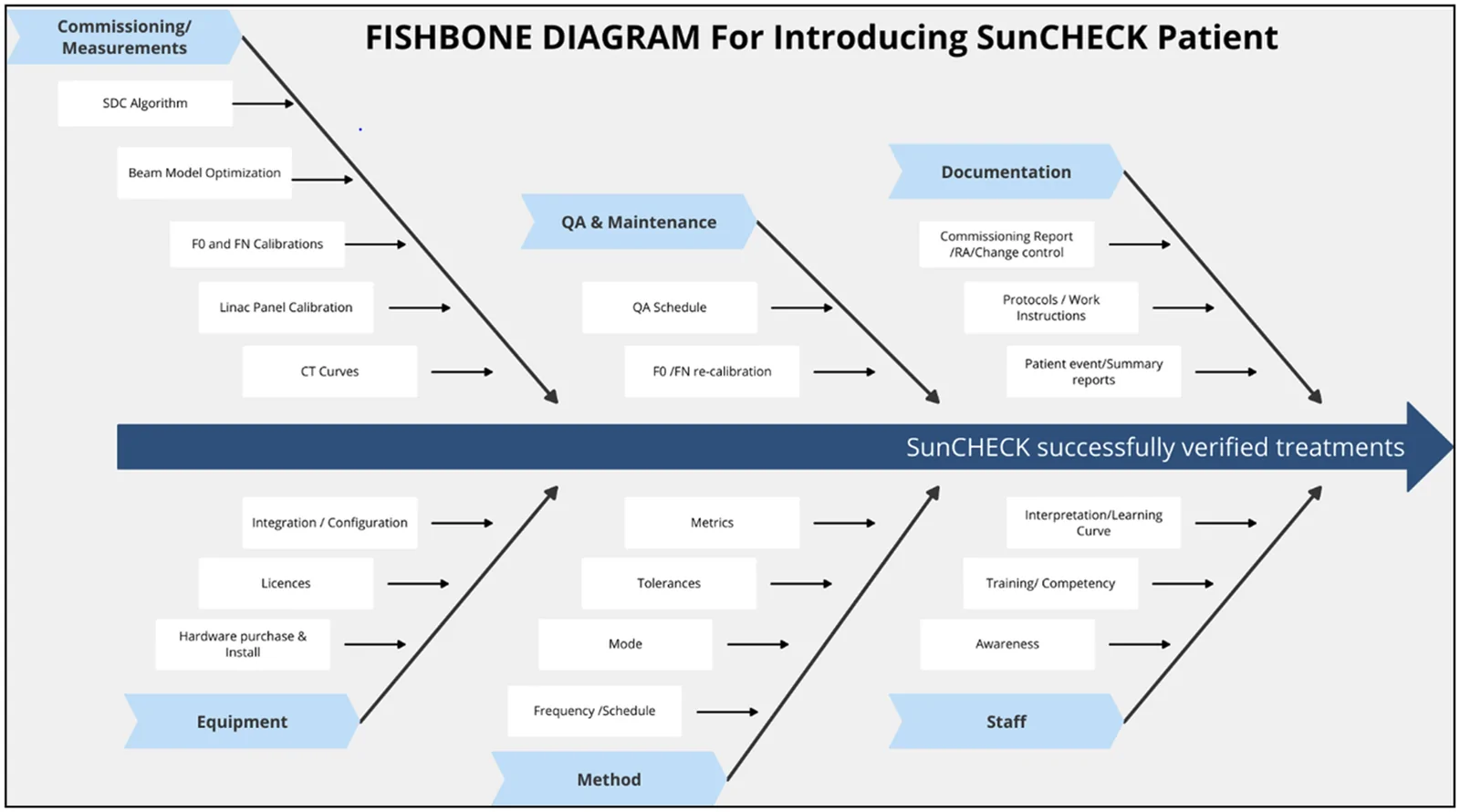
Ishikawa Fishbone diagram outlining the process to introduce SunCHECK Patient (at North West Cancer Center, L’Derry).

The aim of this study was to describe the outcome of a large international survey designed specifically for users of the SunCHECK platform, to determine if there was consensus on how the system is configured among existing real-world users, and the criteria and tolerances applied. The aims of the questions were (1) to provide benchmarking data for current SunCHECK Patient users and (2) to provide guidance for new users. The survey covered the 3 system modules described above: DoseCHECK, F0, and FN.

## Methods

A consortium was formed of SunCHECK Patient users with a breadth of application experience. A survey was developed in SurveyMonkey (Momentive Inc. San Mateo, CA, USA). The questions were designed to minimize free text for ease of analysis, but each included a text or comment option. It covered all aspects of the 3 modules described above and directed users to appropriate sections depending on how they described their use of SunCHECK Patient. The full set of questions is provided in Appendix A1.

The survey was distributed to SunCHECK Patient users in the United Kingdom and Europe (in English only) via local distributors and the UK medical physics mailbase (Physics UK Mailbase: https://www.jiscmail.ac.uk/cgi-bin/webadmin?A0=MEDICAL-PHYSICS-ENGINEERING). Reminders were sent out twice following the initial distribution. Responses were collected between March 7, 2022 and May 23, 2022. Only one response per department was accepted.

Within each module a structured approach to assessing respondent consensus was applied to these data, and cross tabulation was utilized to explore potential influencing factors.

## Results

### General settings

In total 64 responses were received from 18 countries. Of the respondents, 42 described their experience with SunCHECK Patient as either “still commissioning,” “new user,” or “competent user.” The remaining 22 considered themselves “experienced” or “very experienced.” Experienced users were more likely to use the SDC calculation as the 3D reference dose for F0 (61%) and FN (73%) events, while less experienced users were more likely to use the TPS dose as reference (Figure 2, F0 71%, FN 58%). Centers in the United Kingdom were more likely to use SDC as the reference (F0 64%, FN 80%) than centers outside the United Kingdom (F0 34%, FN 48%).

**Figure 2:**
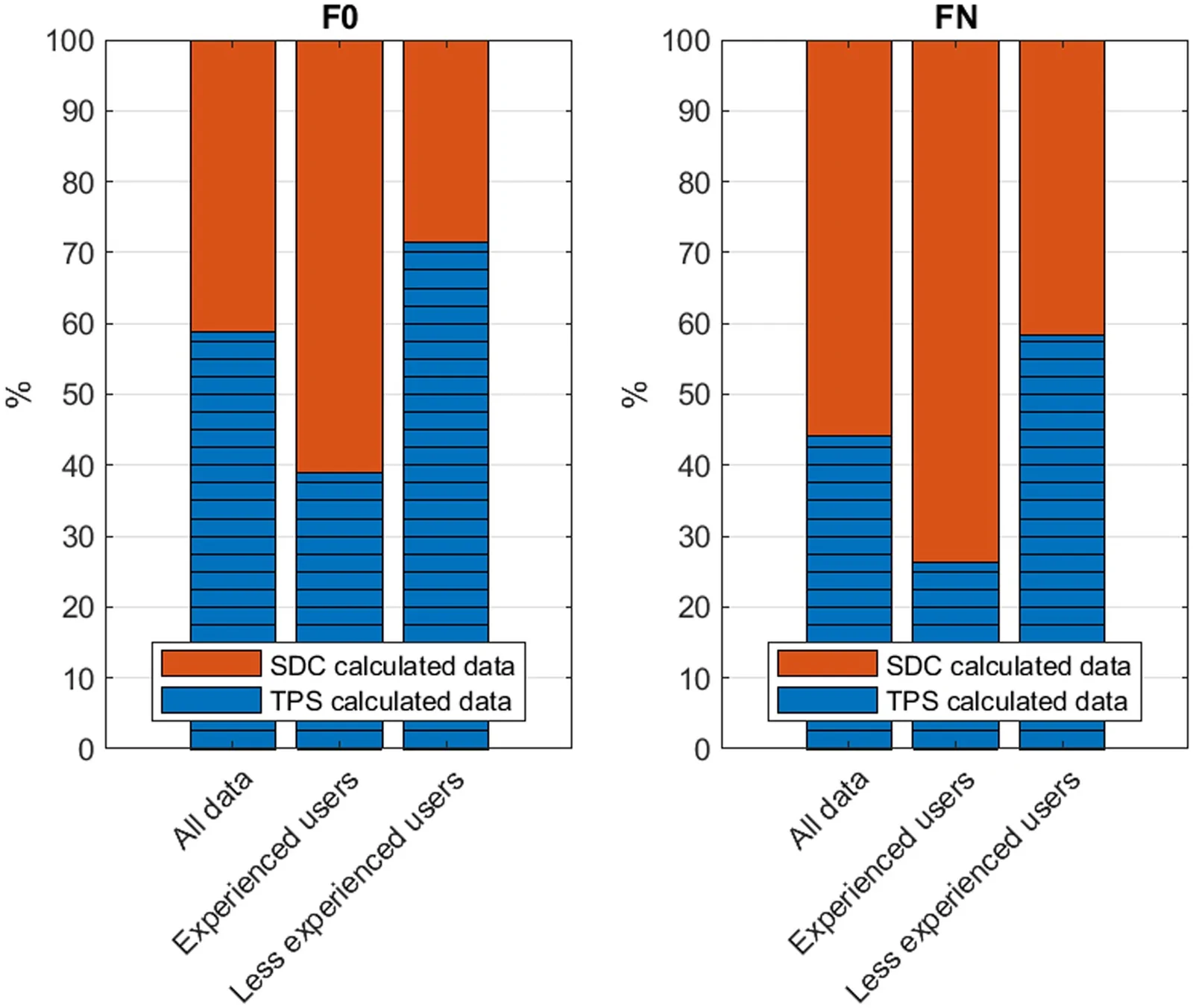
Relative proportions of users using TPS data or SDC calculated data, for F0 or FN, broken down into user experience level with SunCHECK Patient. Percentages are determined for each module, F0 or FN, and within each grouping of users.

A custom beam model is selected by 73% of users, while 27% use the generic model provided by the vendor.^16^ There was no strong vendor-dependent difference observed in model selection (74% of Varian users using custom models; 68% of Elekta users using custom models). Of those who indicate their main intent for FN is to detect clinically significant errors, 91% are using a custom model.

Universal templates are applied by 60% of the 58 users who use DoseCHECK: 60% for F0 and 65% for FN events (Figure 3). Of the 4 users who apply separate warning and action tolerance levels, 3 do so using universal templates.

**Figure 3:**
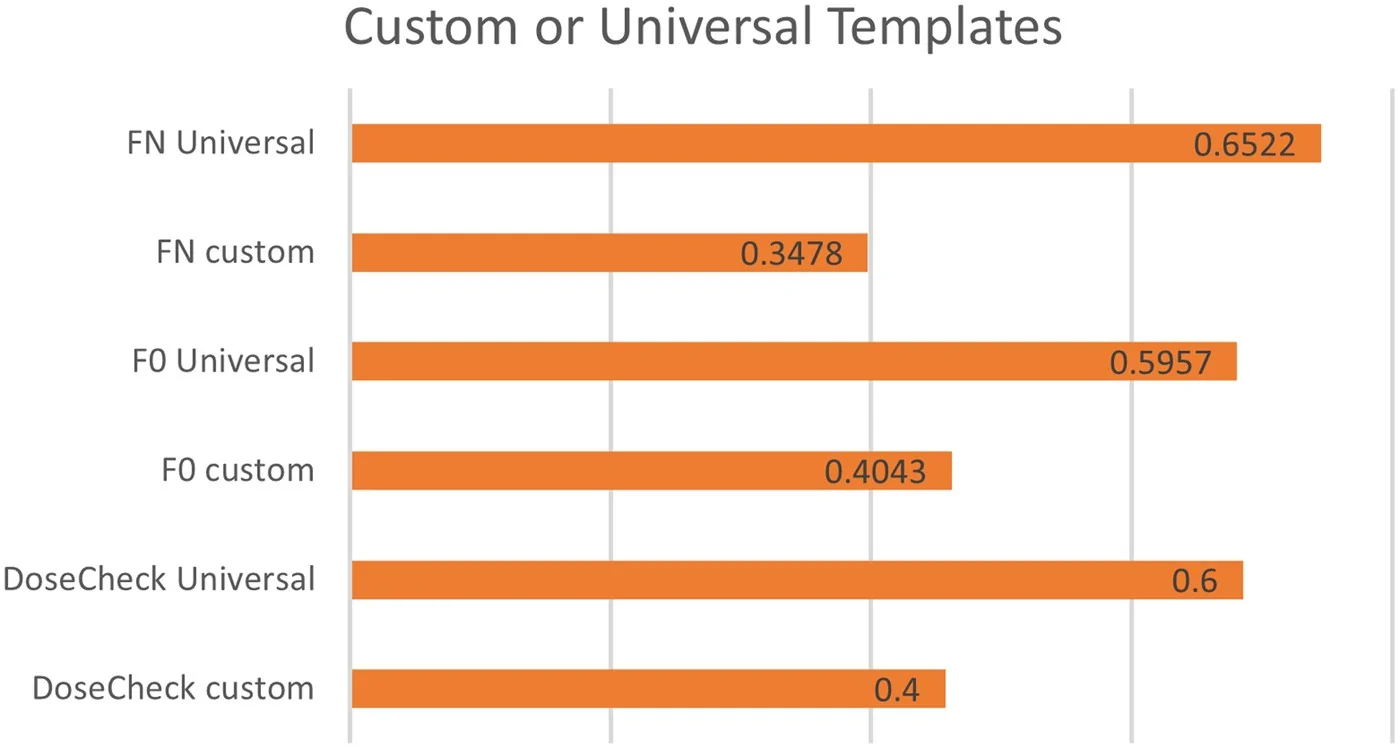
Proportions of users choosing custom templates or universal templates for analysis during F0, FN, and DoseCHECK events. Percentages of either universal or custom are determined for each event.

Global gamma normalization is applied by 88% of DoseCHECK users, 86% of F0 users and 91% of FN users. A threshold of 10% is applied by 88% of DoseCHECK users, 86% of F0 users, and 78% of FN users. Two users indicated different settings depending on the treatment site.

Eight DoseCHECK users and 4 F0 and FN users apply separate action and warning level tolerances. Due to the small numbers no consensus was reached on the warning level criteria.

### DoseCHECK

A total of 58 centers who responded use DoseCHECK for independent treatment plan verification: 40 for all photon plans and 18 for some photon plans. The TPS algorithm types reported were type A (*n* = 2), type B (*n* = 35), and type C (*n* = 28). All respondents that indicated using larger gamma criteria of 7% and/or 5 mm for non-stereotactic sites (*n* = 3) were using type B TPS algorithms.

Of the 22 users using dose to medium (*D*_m_) calculations in their TPS only 27% make a manual dose to medium (*D*_m_) correction in SunCHECK.^17^ There is no correlation in the DoseCHECK tolerances applied by centers reporting *D*_m_ or dose to water.

There was general congruence of criteria and tolerances applied for various treatment types (full data listed in Appendix A1). The data summarized in Table 1 represent the criteria applied by the significant majority of respondents. For all sites, at least 30% of users do not use point dose as a pass or fail criterion. Of those users that do, the majority apply a 3% tolerance. Five users apply a criterion in cGy rather than a percentage. A gamma dose criterion of 3% was most common across all sites (*n* = 21/27, 21/29, 22/29, 21/29 for breast/pelvis/H&N/lung). A distance criterion of 3 mm was most commonly applied (*n* = 19/27, 15/29, 17/29, 17/29 for breast/pelvis/H&N/lung). Over 30% of users applied a distance criterion of 2 mm for H&N (*n* = 10/29), pelvis (12/29), and thorax cases (10/29). For SABR or SRS cases there was no consensus among responses for distance criterion with 1 mm (*n* = 7/24 or 8/19), 2 mm (*n* = 9/24 or 4/19) or 3 mm (*n* = 7/24 or 6/19) being applied.

**Table 1:** Most common DoseCHECK criteria for each metric and treatment site, based on 58 responses.

Treatment site	Point dose	Gamma criteria	Pass rate	Gamma criteria applied to
Breast, 3D conformal, palliative 3D, palliative VMAT	3%	3%/3 mm	95%	Body
Pelvis, head and neck, thorax	3%	3%/2 mm or 3%/3 mm	95%	Body
SABR/SRS	3%	3% [1, 2, or 3 mm]	95%	Body

### Fraction zero

Five centers use F0 for PSQA of all photon plans, including palliative; 14 centers use F0 for all intensity modulated radiation therapy (IMRT) and volumetric modulated arc therapy (VMAT) photon plans while 15 use it for some photon PSQA. F0 was not used at all by 16 centers and 7 did not respond. Of the 34 centers using F0, 79% apply the calibrated dose mode to analyze integrated images in 2D—further details are provided in Figure 4. Only 5 users are performing 3D analyses using log files, 2 of those are not collecting 2D images.

**Figure 4:**
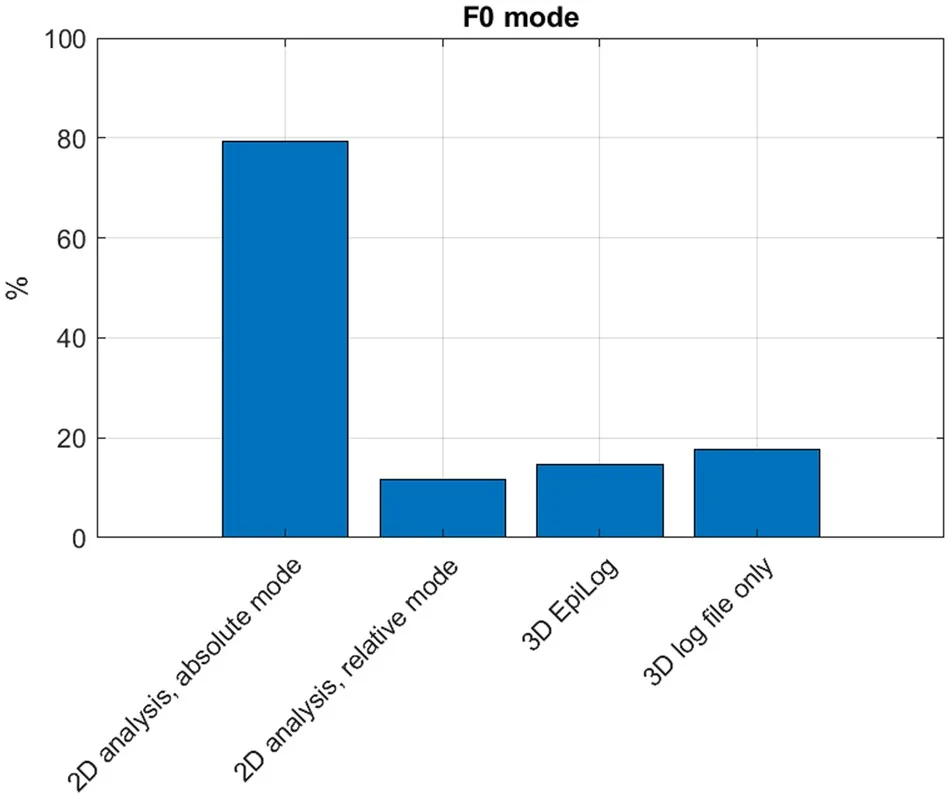
Relative proportions of mode of measurement for F0 events (PSQA). Percentages were calculated for each of 2D and 3D analysis, across all respondents (not all respondents use all modes, hence the values do not add up to 100%).

For all sites, most centers do not use point dose as a pass or fail criterion for F0. Of those centers that do, the criteria employed were 3% or an absolute limit in cGy. There was no consensus on point dose criteria for SRS or SABR plans, which had one response each indicating 2%, 3%, 4%, and cGy criteria.

There was strong consensus for the gamma criteria applied in 2D analysis. For all sites it was either 3%/3 mm (*n* = 8/13, 7/13, 8/14, 7/13 for breast, pelvis, H&N, lung) or 3%/2 mm (*n* = 4/13, 6/13, 6/14, 5/13 for breast, pelvis, H&N, lung), as listed in Table 2, with a pass rate tolerance of 95%. The majority of users do not use 3D gamma analysis, but when used it is applied within the whole body structure.

**Table 2:** Consensus F0 criteria for different metrics and treatment sites, based on 19 responses.

Treatment site	Point dose	2D gamma criteria	Pass rate	3D gamma criteria	Gamma criteria applied to
Breast, 3D conformal, palliative VMAT	3% or cGy	3%/3 mm	95%	3%/2 mm	Body
Palliative 3D	3%	3%/3 mm	95%	3%/3 mm	Body
Pelvis, head and neck, thorax	3%	3%/2 mm or 3%/3 mm	95%	3%/2 mm	Body
SABR SRS		3%/3 mm 3%/2 mm	95%	3%/2 mm	Body

### Fraction N

A total of 31 users indicated use of FN primarily for detecting clinically significant errors (23 users), to detect gross errors (15 users) or to comply with local legislation (4 users).

For SRS or SABR treatments, 52% measure a FN event daily. Otherwise, an FN measurement on Fraction 1 is the most common approach. Other regimes include daily FN for all category one patients or complex plans. This is summarized in Figure 5.

**Figure 5:**
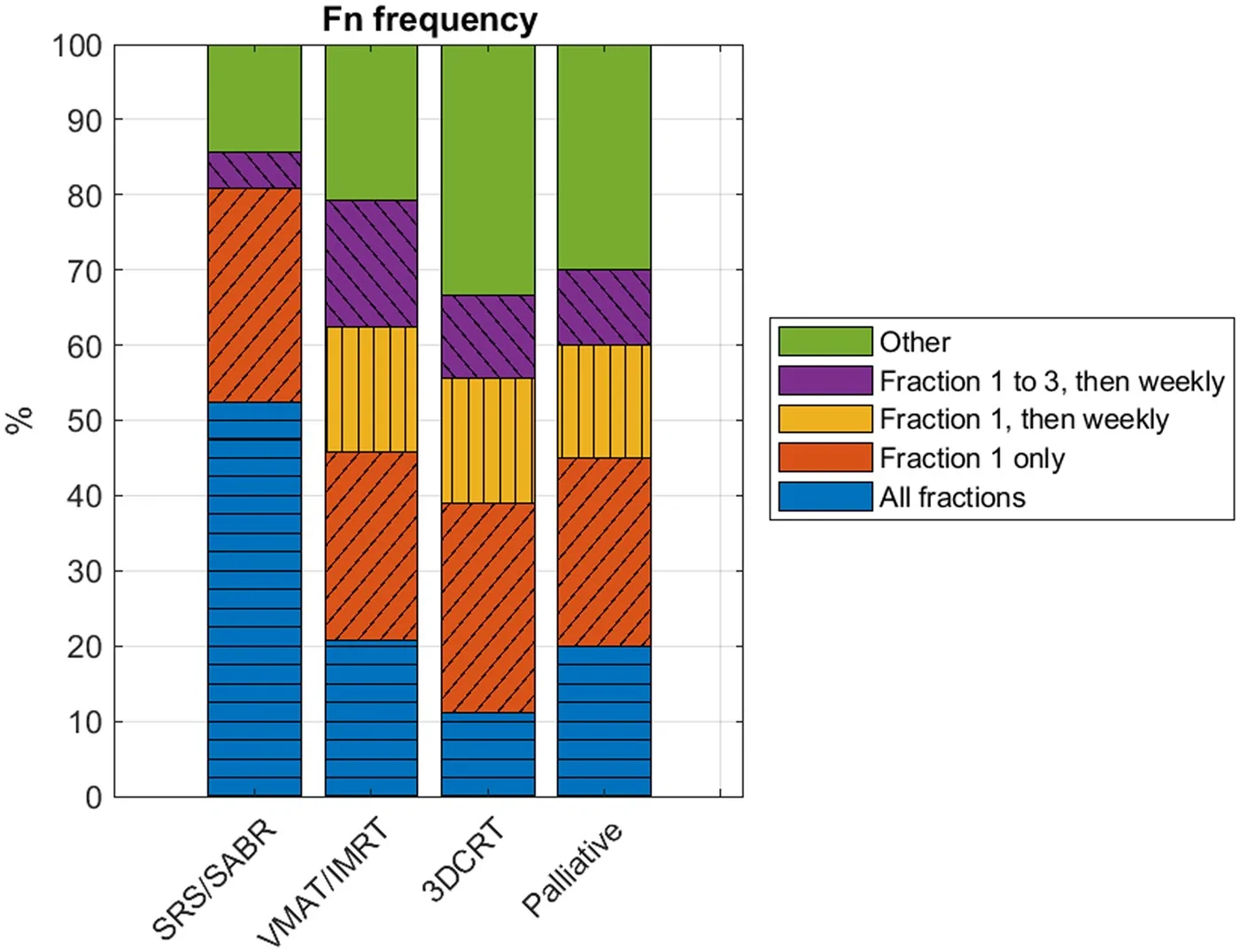
Relative frequencies of measuring FN events for different treatment techniques. The number of responses for each technique was: SRS/SABR *n* = 21; VMAT/IMRT *n* = 23; 3DCRT *n* = 18; Palliative *n* = 20.

No correlation was observed between frequency of measuring FN events and time spent per day on analysis (typically interpretation and understanding of failing fractions). The majority, *n* = 21, spend up to 2 hours per day performing FN analysis. Three spend 3-4 hours per day, however, they do not measure all fractions. They are not the largest centers and 2 use large tolerances to detect gross errors only.

Figure 6 indicates that the majority of FN users utilize the 2D dose mode (the same result as for F0). All centers who perform 3D log-only analysis do so simultaneously with 2D EPID analysis. Five users are calculating 3D Epilog on the CBCT of the day.

**Figure 6:**
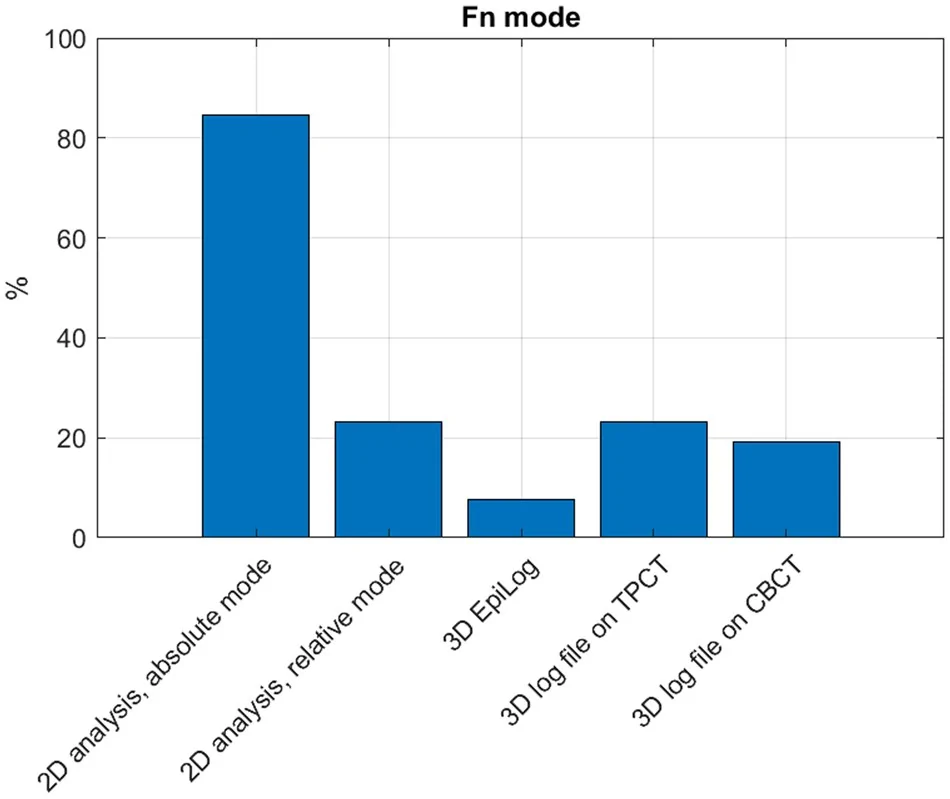
Relative proportions of mode of measurement for FN events (*n* = 26). TPCT refers to treatment planning CT. Percentages were calculated for each of 2D and 3D analysis, across all respondents (not all respondents use all modes, hence the values do not add up to 100%).

The criteria being applied for FN events reflect the split in intent between detecting clinically significant changes or gross errors (shown in Figures 7 and 8). Point dose is not a criterion for 54% of centers. For those with intent to detect clinically significant changes, 2D gamma criteria of 3%/3 mm were generally applied (46/133 select 3%, 50/133 select 3 mm, across all sites). For an intent of detecting gross errors, criteria of either 5% (*n* = 24/133) or 10% (23/133) and a DTA of 5 mm (50/133) were typically applied for 2D analysis. In both instances, the majority (*n* = 8) apply a pass rate of 95%, 4 apply a pass rate of 90%, and 1 uses 85%. Table 3 summarizes the most frequently applied criteria for FN.

**Figure 7:**
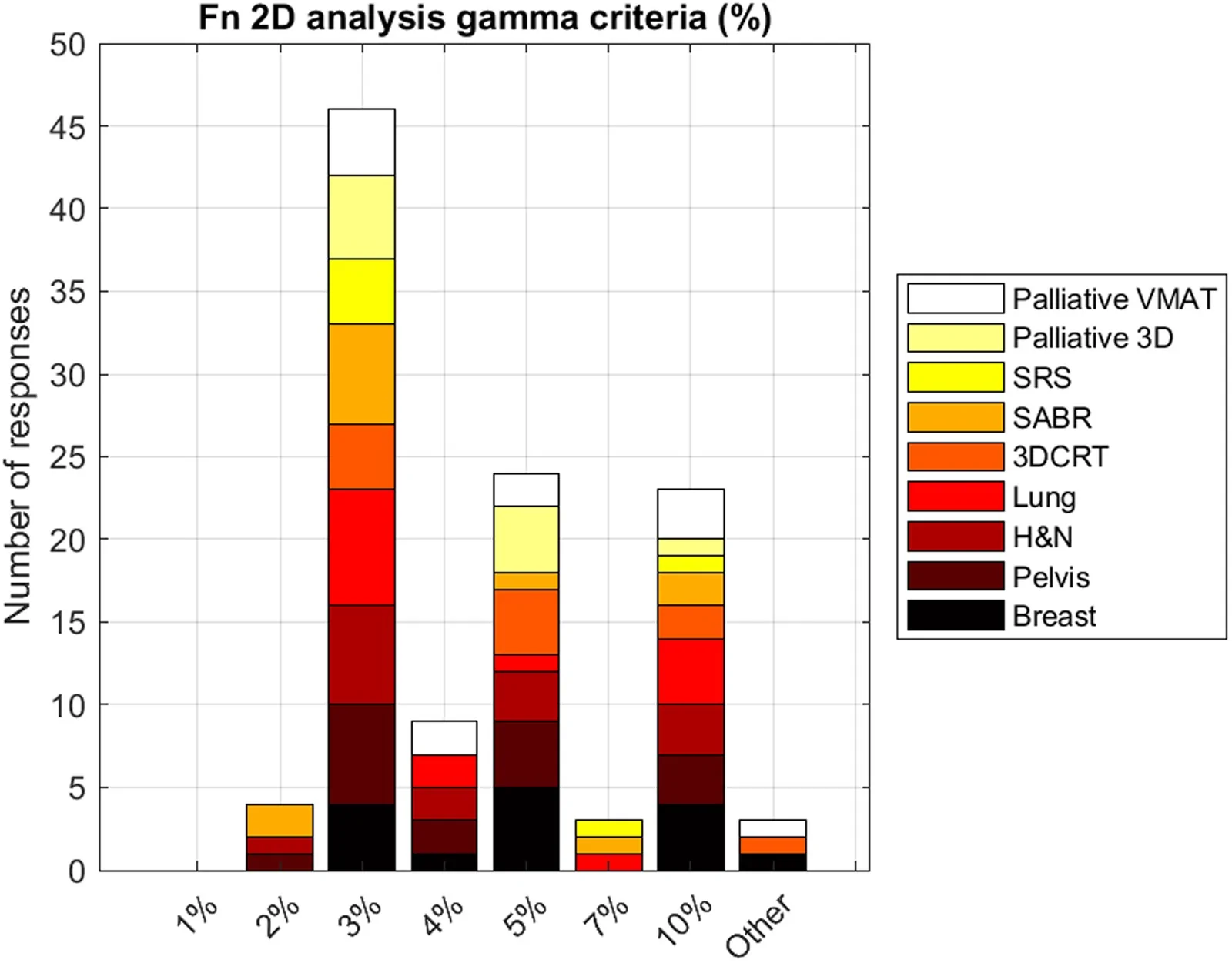
Gamma dose criteria applied for FN events, broken down into different treatment sites or treatment techniques.

**Figure 8:**
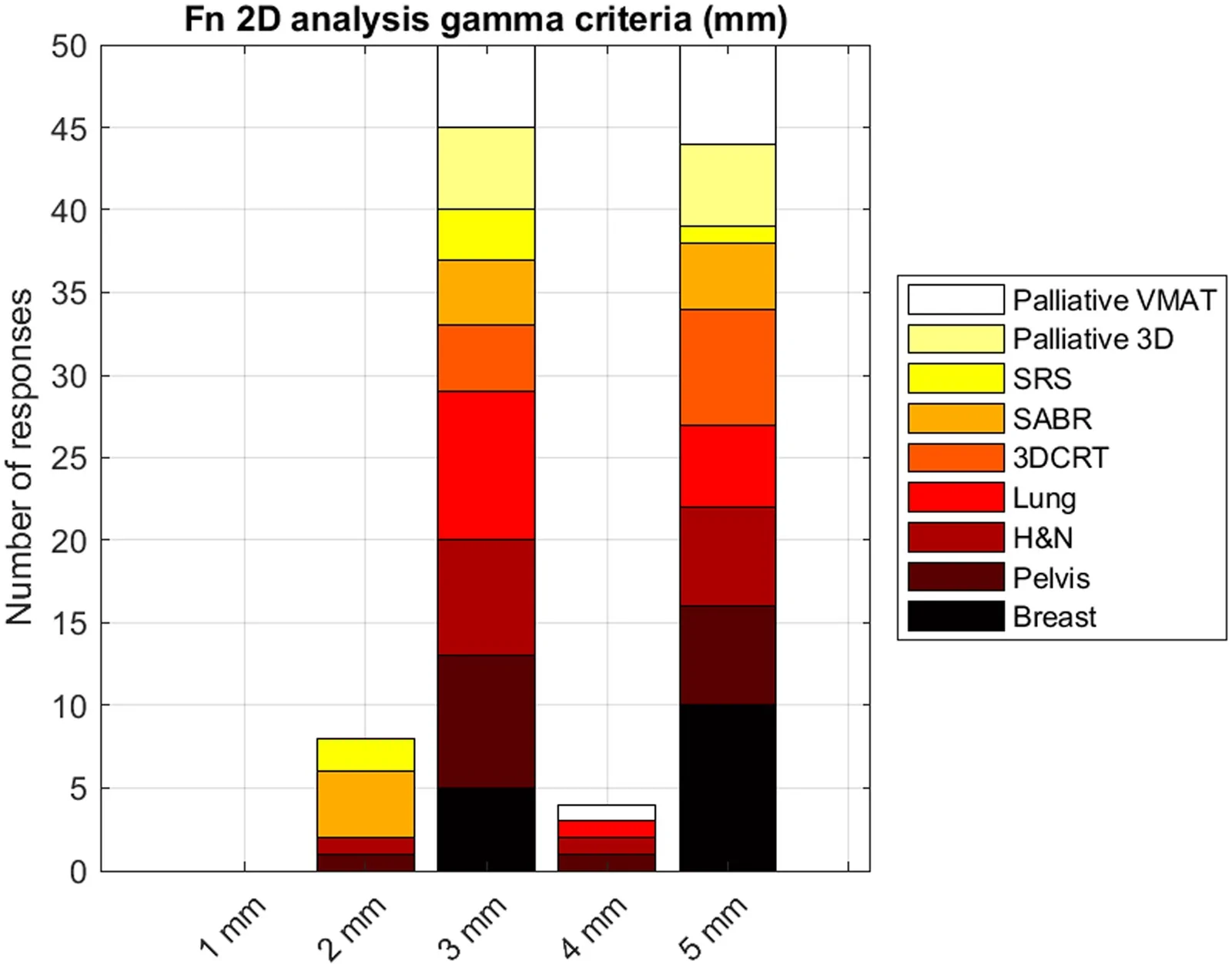
Gamma distance criteria applied for FN events, broken down into different treatment sites or treatment techniques.

**Table 3:** Most frequently applied FN criteria for different metrics and treatment sites.

Treatment site	Point dose	2D gamma criteria	Pass rate, 2D and 3D	3D gamma criteria	Gamma criteria applied to
Breast, 3D conformal, palliative VMAT, palliative 3D	No consensus.	3%/3 mm or 5%/5 mm	95%	3%/3 mm	No consensus
Pelvis, head and neck, thorax		3%/3 mm or 10%/5 mm			
SABR SRS		3% 2 mm or 3 mm or 5 mm			

At least 38% of users do not apply pass or fail criteria on the 3D analysis. For those that do, gamma criteria of 3%/3 mm (23/56) or 10%/5 mm (15/56), are applied according to intent together with a pass rate of 95%. There is no consensus on applying the 3D gamma criteria to the whole body (*n* = 3), the PTV (*n* = 2), or multiple organs (*n* = 2).

Respondents were asked to rank the most common type of error detected with FN (with 1 the most common). The most common are anatomical variations (20%), setup (17%), and immobilization errors (15%). False positives (errors in SNC) accounted for 12%, while 11% had an unidentified cause (Figure 9). The ranking remained approximately the same for all measurement frequencies and tolerances applied.

**Figure 9:**
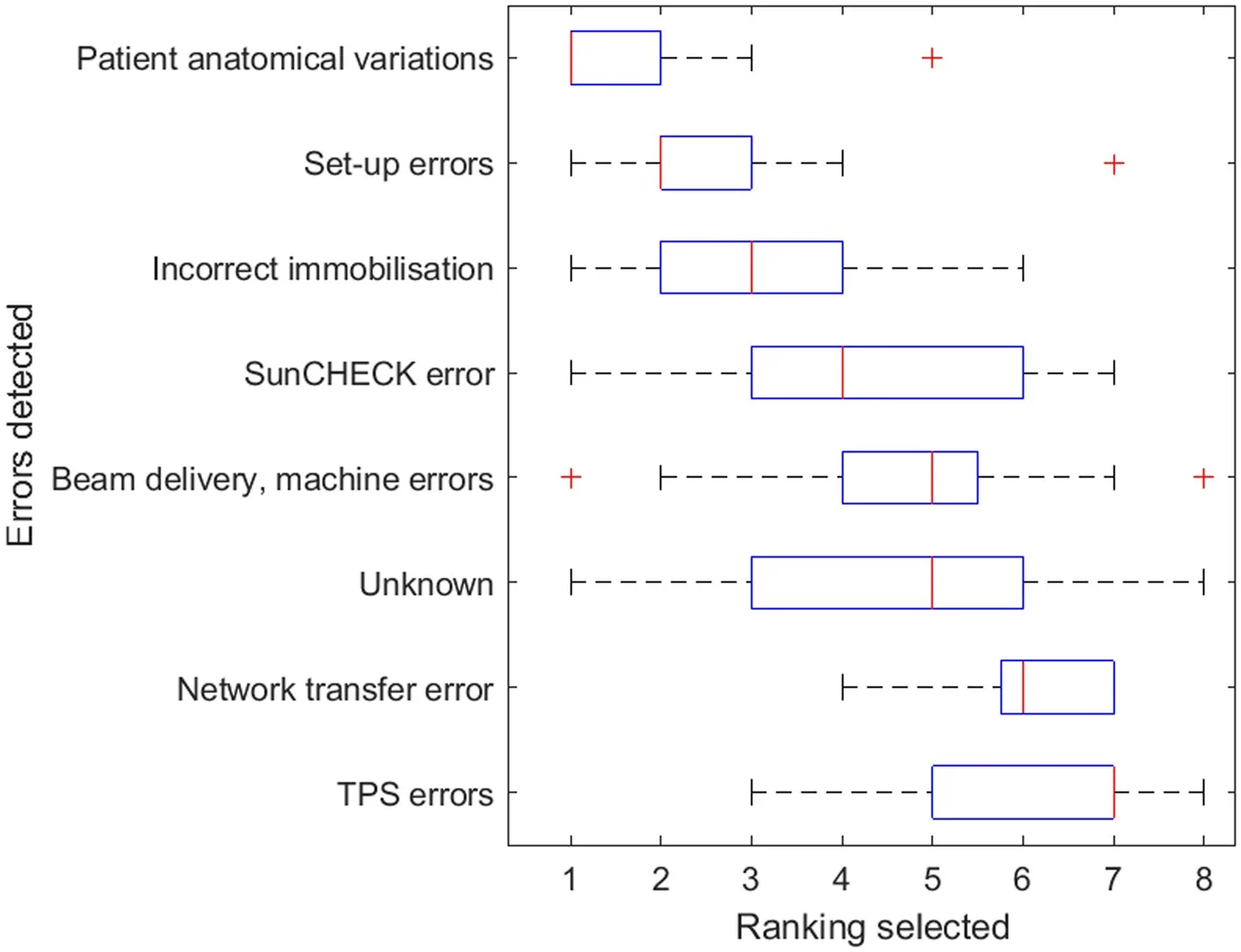
Ranking of most commonly detected treatment errors through analysis of FN measurements, as reported by users.

Almost two-thirds (*n* = 21) of centers indicated they would perform more IVD if they had adequate staff or time, 6 others said they would need increased budget or licenses. Six users feel they perform sufficient IVD measurements (one of whom performs daily measurements).

### Calibrations

At least 62% of users perform F0 and FN software calibrations annually or less. Elekta (Stockholm, Sweden) linac users are likely to calibrate more often than Varian linac users (hardware calibrations of the EPID panel are recommended annually by Elekta and 4-monthly by Varian). QA of the F0 or FN calibrations (independent of EPID QA) is not performed by 47% of users. Of the 53% performing QA, 33% perform this routinely, 20% ad hoc. Several comments indicate that the QA takes the form of performing measurements of a standard plan.

There is strong consensus for both F0 and FN calibrations to apply the gamma criteria with global normalization, 10% threshold at 3%/3 mm and a 95% passing rate. Comments reflect that a 98% pass rate is achievable, except for fields at the edge of the panel. Visual inspection of the calibration fields is also carried out.

## Discussion

The SunCHECK Patient platform provides a range of automated options for performing and analyzing TPS plan verifications, PSQA, and online treatment monitoring. It is highly customizable, yet there appears to be significant commonality of approach from users. Linac vendor and TPS have little influence on the analysis criteria applied.

Custom beam models were found to be more commonly used than generic models. These user-specific custom models are generated by providing beam data and treatment plans to Sun Nuclear, and were reported to be preferred because they achieve closer agreement between the TPS and SunCHECK. Other respondents preferred the generic model for increased independence. Experienced users are more likely to use the SDC calculated data as the reference for 3D F0 and FN analysis; this reduces false failures by removing discrepancies between the TPS and SDC algorithms already acknowledged in the DoseCHECK event.

There is a minor bias for centers with Varian linacs to be more likely to use custom analysis templates. The possible historical explanation for this could be that universal templates were not available in the original versions of SunCHECK, which was initially released for Varian users. Despite this, few centers are using warning level criteria for any events. Universal templates are recommended for new users to efficiently configure and commission the system.

As stated in the introduction, the SDC algorithm is used for dose calculations within SunCHECK. The recommendation of AAPM Task Group 329^17^ is that although SDC does vary the energy absorption coefficients based on electron density, the soft-tissue density range is equivalent to dose-to-water and therefore a *D*_m_ correction should be applied. Therefore, if the TPS is reporting *D*_m_ then users should take care to understand these implications on their local system.

The gamma normalization and threshold used in most centers concurs with the recommendations of AAPM TG218 for IMRT QA. Some experienced users utilize higher thresholds, particularly in FN events, to reduce investigations of gamma discrepancies in low-dose regions.

The lack of point dose as a pass or fail criterion in many centers reflects the Euratom recommendation^3^ that a single point check is not appropriate for dynamic deliveries. In general, 3D gamma analysis is applied instead for the whole body region.

Bossuyt et al^18^ suggested that a common set of parameters is not feasible for clinical practice. However, for both DoseCHECK and F0 events, there is homogeneity among users on the gamma criteria applied of either 3%/3 mm or 3%/2 mm for all sites (the larger generally being applied for palliative and breast plans). The recommendations of TG218 are to apply 3%/2 mm criteria and a universal tolerance >95% pass rate for IMRT PSQA; approximately half of respondents in this survey concur with this recommendation.

The FN gamma criteria depend on the motivation behind performing the FN measurements. Centers are either intending to detect clinically significant errors or gross errors. Despite the 2 different aims there was no difference between how frequently the FN measurements are performed or how much time is spent daily on analysis. In a multicenter study of Epilog EIVD, Esposito et al^19^ also found that centers applied a range of criteria, however such differences were attributed to different treatment sites or techniques and a lack of guidelines. In the present survey it seems clear that there are 2 distinct approaches.

In determining suitable FN criteria to apply, it is important to consider the frequency of failing results so that the workload created is manageable and failures can be acted on promptly. Users should also consider tradeoffs and risks arising from alert fatigue.^20^ By retrospectively analyzing data it is possible to vary the criteria, observe the change in passing rate, and then select the criteria that gives the desired failure rate. Sensitivity analysis may be a suitable approach to determine tolerances that lead to specific failure rates. Another approach is to assess the data empirically, using a common starting point (typically 3%/3 mm) and varying for different anatomical sites as required—such an approach has been utilized by Bossuyt et al.^18^ Fuangrod et al.^21^ showed that Statistical Process Control methods can be used to objectively assess the passing rates and set the lower control limits for daily EPID online treatment monitoring.

Recalculation on the daily cone beam CT was not common. Reasons for this are not clear, but it is speculated that it could be due to known limitations of SunCHECK. There is no algorithm for deformable image registration, and hence the structure set is applied as planned to the CBCT. Also, in the software version available at the time of the survey, only a single Hounsfield Unit to electron or mass density calibration was available per linac, leading to issues when using CBCTs acquired with varying scan parameters. However, since the survey was conducted, the software has been updated to allow for multiple calibrations.

As shown in Figure 9, the errors most frequently detected by the SunCHECK users are patient and setup-related, rather than systematic. This concurs with findings reported by Bossuyt et al^18^ and would endorse a need for frequent monitoring of treatment fractions. Analysis of the 2D data can be difficult to interpret and therefore it may have been expected that users with greater experience would spend less time on analysis, however, this was not reflected in the survey results. Most centers have a desire to perform more EIVD measurements, but lack of time or staff remains a barrier despite the automation of the platform.

New users may wish to consider an initial rollout with lower frequency measurements and/or applying more generous tolerances. This would ensure expedient analysis of the resulting failures and inspire confidence in the platform across the service.

Most users are not performing routine QA of their F0 and FN calibrations. The vendor recommends establishing a local QA process, such as a standard plan, and we further endorse this. Imaging panel calibrations can impact the SunCHECK calibrations and QA is recommended after panel maintenance to verify the F0 and FN calibrations remain valid. Generally, annual F0 and FN calibrations are sufficient.

In general, there is some alignment with the consensus tolerances and settings applied and the default settings in the system, however the vendor does not specify the use of any specific criteria.

The following suggestions would help new users establish their system configuration in line with the majority of the SunCHECK community:

- Use SDC calculated dose as the reference dose for 3D F0 and FN events;
- Use universal templates to allow warning and out of tolerance levels to be applied simultaneously, and to assist with efficient configuration for new users;
- Apply global gamma normalization with a 10% threshold. A larger threshold can be applied for troubleshooting or to reduce false positives on specific plans;
- Using gamma criteria of 3%/2 mm is an adequate starting point for all photon IMRT and VMAT plans, for DoseCHECK and F0 events;
- Consider clinical intent, resource requirement and alert frequency when configuring FN criteria;
- Only perform FN measurements as frequently as your resources allow such that failures can be promptly investigated;
- Establish a routine QA program for F0 and FN calibrations.

This survey was circulated in English only, which may have been a barrier to participation for some centers. It is limited to a single commercial platform and therefore may not be readily applicable to other systems. It is possible that the homogeneity of settings being applied in the platform could have tended toward the default values.

## Conclusion

This survey has shown that there is consensus among SunCHECK users in terms of tolerances and criteria applied for DoseCHECK and F0. For EIVD events (FN) the investigation tolerances applied vary depending on the clinical intent. The linac vendor, TPS or user experience with SunCHECK have little influence on the applied tolerances.

## Supplementary Material

tqag166_Supplementary_Data
