# Supplementary material for: Results of an international survey of SunCHECK Patient users: consensus and benchmark data for plan verification, pre-treatment quality assurance, and in vivo dosimetry tolerances
Source: Br J Radiol. 2026 Jul 29;99(1187):2326–35. doi: 10.1093/bjr/tqag166 (PMC13687761; doi:10.1093/bjr/tqag166)
Supplement: Supplementary file 1 [file tqag166_supplementary_data.zip › Appendix A1 - Survey Questionnaire.docx]

# Appendix A1: Survey Questionnaire

|  | **Question** | **Answer Choice**  **(blank = free text)** | **Sub-option**  **(if applicable)** |
| --- | --- | --- | --- |
| 1.0 | *What country are you responding from?* |  | |
| 2.0 | *What Cancer Centre are you responding from?* |  | |
| 3.0 | *How would you describe your department's experience level with SunCHECK Patient?* | Still commissioning /New /Competent | |
|  |  | Experienced / Very Experienced | |
| 4.0 | *What  Oncology management system do you have?* | Aria | |
|  |  | Mosaiq | |
| 5.0 | *What linear accelerators do you have (that you use with SunCheck)?* | Varian | |
|  |  | Elekta | |
| 6.0 | *In SunCheck do you use the Generic beam model or a site specific beam model?* | Generic | |
|  |  | Site Specific | |
| 7.0 | *If you use F0 and FN, is your reference data the TPS calculated data or the SDC calcalculated data?* | TPS | F0 |
|  |  |  | FN |
|  |  | SDC | F0 |
|  |  |  | FN |
| 8.0 | *Do you use Universal Metric or Custom Templates?* | Custom | DoseCheck |
|  |  |  | F0 |
|  |  |  | FN |
|  |  | Universal | DoseCheck |
|  |  |  | F0 |
|  |  |  | FN |
| 9.0 | *For Gamma Analysis, what normalisation and threshold do you use?* | Normalisation (Global / Local) | DoseCheck |
|  |  |  | F0 |
|  |  |  | FN |
|  |  | Threshold (10%/ other) | DoseCheck |
|  |  |  | F0 |
|  |  |  | FN |
| 10.0 | *Do you use SNC DoseCHECK as an independent check for photon plans?* | Yes (All or some photon plans) | |
|  |  | No | |
| 11.0 | *What algorithms and medium are you using in your planning system?  (Select all that apply)* | Type A | |
|  |  | Type B | |
|  |  | Type C | |
|  |  | Dose medium | |
|  |  | Dose water | |
| 12.0 | *Do you apply a dose to medium correction in SunCHECK?* | Yes | |
|  |  | No | |
| 13.0 | *What Suspension level  / Failed Gamma criteria do you apply for DoseCHECK analysis?* | See criteria sheet (Appendix A2) | |
| 14.0 | *Separate Action level?* | Yes | |
|  |  | No | |
| 15.0 | *What action level  / warning Gamma criteria do you apply for DoseCHECK analysis?* | See criteria sheet (Appendix A2) | |
| 16.0 | *Do you use PF* | Yes (all or some) | |
|  |  | No | |
| 17.0 | *What mode do you use for F0 QC?* | 2D absolute | |
|  |  | 2D relative | |
|  |  | Cine 3D EpiLog | |
|  |  | Log only 3D | |
| 18.0 | *What Suspension level Gamma criteria do you apply for F0?* | See criteria sheet (Appendix A2) | |
| 19.0 | *Do you use separate Warning / Action tolerances for F0?* | Yes | |
|  |  | No | |
| 20.0 | *What warning level Gamma criteria do you apply for F0?* | See criteria sheet (Appendix A2) | |
| 21.0 | *Do you use FN for In Vivo dosimetry?* | Yes | |
|  |  | No | |
| 22.0 | *How often do you perform FN In Vivo dosimetry?* | SABR | Daily |
|  |  |  | F1 |
|  |  |  | F1 then weekly |
|  |  |  | F1-3 then weekly |
|  |  |  | Other |
|  |  | VMAT/ IMRT | Daily |
|  |  |  | F1 |
|  |  |  | F1 then weekly |
|  |  |  | F1-3 then weekly |
|  |  |  | Other |
|  |  | 3D CRT | Daily |
|  |  |  | F1 |
|  |  |  | F1 then weekly |
|  |  |  | F1-3 then weekly |
|  |  |  | Other |
|  |  | PALLIATIVE | Daily |
|  |  |  | F1 |
|  |  |  | F1 then weekly |
|  |  |  | F1-3 then weekly |
|  |  |  | Other |
| 23.0 | *To determine how much resource has been invested in your EPID In Vivo dosimetry please answer the following in relation to FN dosimetry:* | <1 hr | |
|  |  | 1-2 Hr | |
|  |  | 3-4 Hr | |
|  |  | >4 Hr | |
| 24.0 | *What is your main intent when performing FN In Vivo dosimetry?* | Gross error detection | |
|  |  | Detect clinically significant variations | |
|  |  | Legislation | |
|  |  | Other | |
| 25.0 | *How do you perform FN In Vivo dosimetry? (Select all that apply)* | 2D absolute | |
|  |  | 2D relative | |
|  |  | Cine 3D EpiLog | |
|  |  | Log only 3D | |
|  |  | CBCT | |
| 26.0 | *What Suspension level / Failure Gamma criteria do you apply for FN? (If N/A, leave blank)* | See criteria sheet (Appendix A2) | |
| 27.0 | *Do you use separate Warning / Action tolerances for FN?* | Yes | |
|  |  | No | |
| 28.0 | *What warning level Gamma criteria do you apply for FN? (If N/A leave blank)* | See criteria sheet (Appendix A2) | |
| 29.0 | *What types of errors have you detected with your In Vivo dosimetry?  Please rank from most to least frequent* | Set Up errors | |
|  |  | Anatomical variations | |
|  |  | Incorrect Immobilization | |
|  |  | Beam delivery / machine error | |
|  |  | TPS error | |
|  |  | Network transfer error | |
|  |  | Error in SNC | |
|  |  | Unknown | |
| 30.0 | *Would you ideally like to perform more In Vivo Dosimetry?* | Yes if more staff | |
|  |  | Yes if more budget | |
|  |  | No we have right balance | |
|  |  | No we do Too much | |
|  |  | Other | |
| 31.0 | *How often do you perform F0 / FN calibrations?* | F0 | Less than annual |
|  |  |  | Annual |
|  |  |  | More than annual |
|  |  |  | After panel calibrations |
|  |  |  | Other |
|  |  | FN | Less than annual |
|  |  |  | Annual |
|  |  |  | More than annual |
|  |  |  | After panel calibrations |
|  |  |  | Other |
| 32.0 | *Do you perform QA on your F0 / FN calibrations? If yes please comment on what QA you perform, e.g. standard plans* | Yes, routine QC | |
|  |  | Yes, ad hoc QC | |
|  |  | No | |
| 33.0 | *What gamma criteria do you apply to F0 / FN template calibrations?* | Gamma normalisation global / local | F0 |
|  |  |  | FN |
|  |  | Gamma threshold 10% / other | F0 |
|  |  |  | FN |
|  |  | Gamma Criteria 3%/3mm / Other | F0 |
|  |  |  | FN |
|  |  | Gamma pass 95% / other | F0 |
|  |  |  | FN |
