# Supplementary material for: Results of an international survey of SunCHECK Patient users: consensus and benchmark data for plan verification, pre-treatment quality assurance, and in vivo dosimetry tolerances
Source: Br J Radiol. 2026 Jul 29;99(1187):2326–35. doi: 10.1093/bjr/tqag166 (PMC13687761; doi:10.1093/bjr/tqag166)
Supplement: Supplementary file 1 [file tqag166_supplementary_data.zip › Appendix A2 - Suspension table.docx]

# Appendix A2: Warning/Suspension Levels

| **Question Set** | **Anatomical site**  **(Criteria selection for each)** | **Scoring** | **Criterion** |
| --- | --- | --- | --- |
| *What Suspension level  / Failed Gamma criteria do you apply for* ***DoseCHECK*** *analysis?* | **Breast \| Pelvis \| H&N \| Lung Thorax \| 3DCRT \| SABR \| SRS \| Palliative (3D) \| Palliative (VMAT)** | **Point Dose (%)** | 2 |
|  |  |  | 3 |
|  |  |  | 4 |
|  |  |  | 5 |
|  |  |  | cGy |
|  |  |  | Other |
|  |  | **3D Gamma (%)** | 2 |
|  |  |  | 3 |
|  |  |  | 4 |
|  |  |  | 5 |
|  |  |  | 7 |
|  |  |  | 10 |
|  |  |  | Other |
|  |  | **3D Gamma (mm)** | 1 |
|  |  |  | 2 |
|  |  |  | 3 |
|  |  |  | 4 |
|  |  |  | 5 |
|  |  |  | 7 |
|  |  |  | 10 |
|  |  |  | Other |
|  |  | **3D % Gamma Pass** | 98 |
|  |  |  | 95 |
|  |  |  | 90 |
|  |  |  | 85 |
|  |  |  | other |
|  |  | **Gamma Overall or Organ?** | Body |
|  |  |  | PTV |
|  |  |  | Multiple organs |
|  |  |  | Other |
| *What Suspension level  / Failed Gamma criteria do you apply for* ***F0*** *analysis?* | **Breast \| Pelvis \| H&N \| Lung Thorax \| 3DCRT \| SABR \| SRS \| Palliative (3D) \| Palliative (VMAT)** | **Point Dose (%)** | 2 |
|  |  |  | 3 |
|  |  |  | 4 |
|  |  |  | 5 |
|  |  |  | cGy |
|  |  |  | Other |
|  |  | **2D Gamma (%)** | 2 |
|  |  |  | 3 |
|  |  |  | 4 |
|  |  |  | 5 |
|  |  |  | 7 |
|  |  |  | 10 |
|  |  |  | Other |
|  |  | **2D Gamma (mm)** | 1 |
|  |  |  | 2 |
|  |  |  | 3 |
|  |  |  | 4 |
|  |  |  | 5 |
|  |  |  | 7 |
|  |  |  | 10 |
|  |  |  | Other |
|  |  | **2D % Gamma Pass** | 98 |
|  |  |  | 95 |
|  |  |  | 90 |
|  |  |  | 85 |
|  |  |  | Other |
|  |  | **3D Gamma (%/mm)** | 2%/1mm |
|  |  |  | 2%/2mm |
|  |  |  | 3%/1mm |
|  |  |  | 3%/2mm |
|  |  |  | 3%/3mm |
|  |  |  | 4%/3mm |
|  |  |  | 4%/4mm |
|  |  |  | 5%/5mm |
|  |  |  | 10%/5mm |
|  |  |  | Other |
|  |  | **3D % Gamma Pass** | 98 |
|  |  |  | 95 |
|  |  |  | 90 |
|  |  |  | 85 |
|  |  |  | Other |
|  |  | **Gamma Overall or Organ?** | Body |
|  |  |  | PTV |
|  |  |  | Multiple organs |
|  |  |  | Other |
| *What Suspension level  / Failed Gamma criteria do you apply for* ***FN*** *analysis?* | **Breast \| Pelvis \| H&N \| Lung Thorax \| 3DCRT \| SABR \| SRS \| Palliative (3D) \| Palliative (VMAT)** | **Point Dose (%)** | 2 |
|  |  |  | 3 |
|  |  |  | 4 |
|  |  |  | 5 |
|  |  |  | cGy |
|  |  |  | Other |
|  |  | **2D Gamma (%)** | 2 |
|  |  |  | 3 |
|  |  |  | 4 |
|  |  |  | 5 |
|  |  |  | 7 |
|  |  |  | 10 |
|  |  |  | Other |
|  |  | **2D Gamma (mm)** | 1 |
|  |  |  | 2 |
|  |  |  | 3 |
|  |  |  | 4 |
|  |  |  | 5 |
|  |  |  | 7 |
|  |  |  | 10 |
|  |  |  | Other |
|  |  | **2D % Gamma Pass** | 98 |
|  |  |  | 95 |
|  |  |  | 90 |
|  |  |  | 85 |
|  |  |  | Other |
|  |  | **3D Gamma (%/mm)** | 2%/1mm |
|  |  |  | 2%/2mm |
|  |  |  | 3%/1mm |
|  |  |  | 3%/2mm |
|  |  |  | 3%/3mm |
|  |  |  | 4%/3mm |
|  |  |  | 4%/4mm |
|  |  |  | 5%/5mm |
|  |  |  | 10%/5mm |
|  |  |  | other |
|  |  | **3D % Gamma Pass** | 98 |
|  |  |  | 95 |
|  |  |  | 90 |
|  |  |  | 85 |
|  |  |  | Other |
|  |  | **Gamma Overall or Organ?** | Body |
|  |  |  | PTV |
|  |  |  | Multiple organs |
|  |  |  | Other |
